# Editorial: Recent advances in computational modelling of biomolecular complexes

**DOI:** 10.3389/fchem.2023.1200409

**Published:** 2023-04-11

**Authors:** Simón Poblete, Sergio Pantano, Kei-ichi Okazaki, Zhongjie Liang, Kurt Kremer, Adolfo B. Poma

**Affiliations:** ^1^ Instituto de Ciencias Físicas y Matemáticas, Universidad Austral de Chile, Valdivia, Chile; ^2^ Computational Biology Lab, Fundación Ciencia & Vida, Santiago, Chile; ^3^ Institut Pasteur de Montevideo, Montevideo, Uruguay; ^4^ Department of Theoretical and Computational Molecular Science, Institute for Molecular Science, National Institutes of Natural Sciences, Okazaki, Japan; ^5^ Center for Systems Biology, Department of Bioinformatics, School of Biology and Basic Medical Sciences, Soochow University, Suzhou, China; ^6^ Max Planck Institute for Polymer Research, Mainz, Germany; ^7^ Biosystems and Soft Matter Division, Institute of Fundamental Technological Research, Polish Academy of Sciences, Warsaw, Poland

**Keywords:** coarse-grained method, machine learning, multiscale approach, biopolymers, aggregation, GōMartini approach, Martini 3, nanomechanics

The spatiotemporal description of the molecular interactions that rule the biological world poses tremendous challenges to current modeling and molecular dynamics (MD) simulation methods (Pantano, 2022). The deep intricacies of interactions spanning several orders of magnitude in time and space have prompted the scientific community to develop novel methods to enhance our understanding of biomolecular complexes.

**FIGURE 1:**
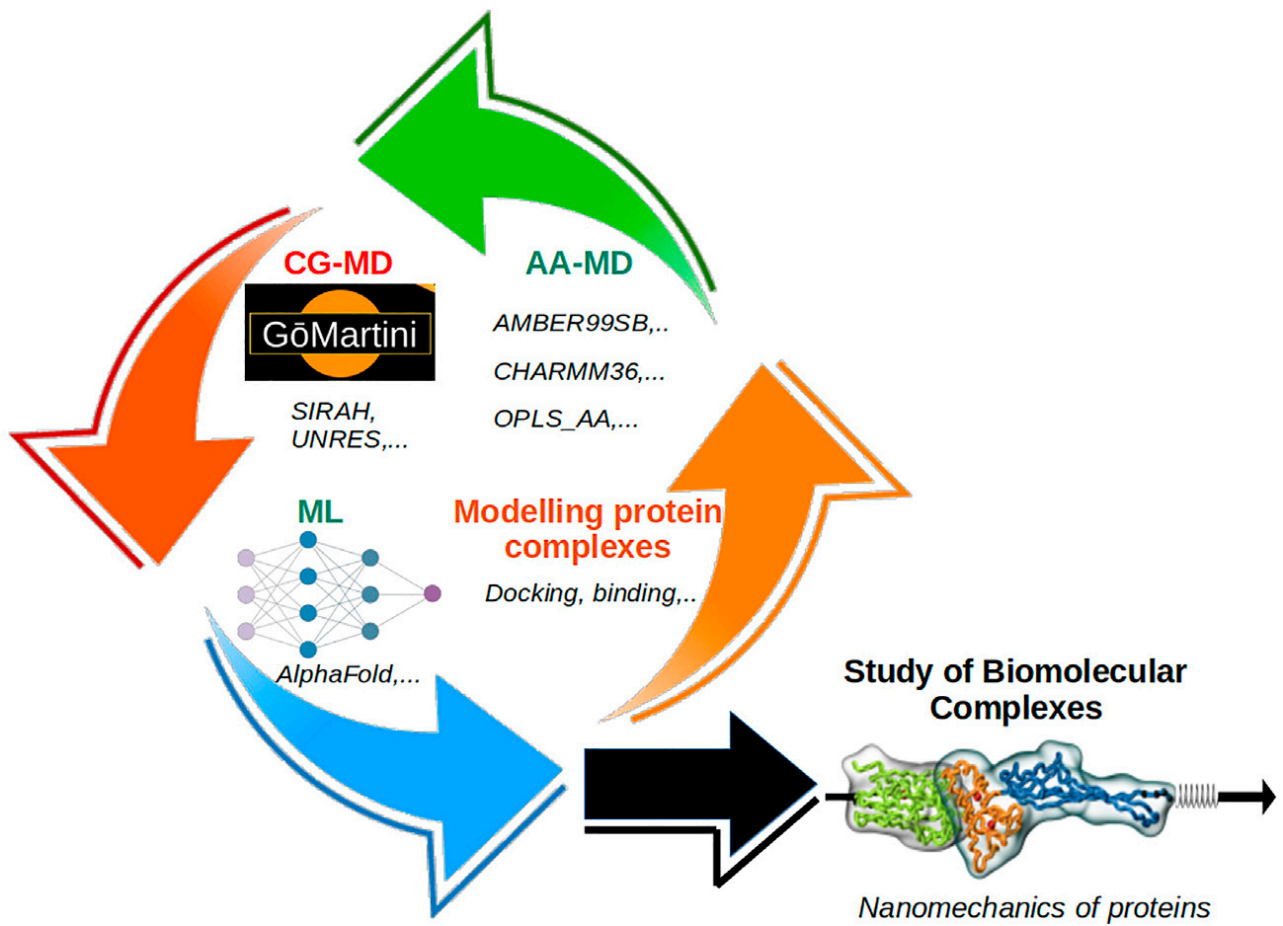
Schematic description of the integration of different methodologies employed in the study of biomolecular complexes: i) all-atom MD, ii) coarse-grained MD, iii) machine learning, and iv) modeling tools. Together they can cope with the study of complex molecular systems, such as biomechanics of protein complexes and other systems at the cellular level.

We present a Research Topic illustrating state-of-the-art applications to study key constituents of biological matter. The modeling of large complexes demands the development of new approaches which are derived based on statistical and thermodynamic principles, such as the case of coarse-grained (CG) methods (Ingólfsson et al., 2022). Some CG studies in this Research Topic deal with the nanomechanics of protein complexes by the GōMartini approach (Liu et al., 2021; Mahmood et al., 2021), the first-ever CG modeling of an entire cell, coupling of different molecular resolutions (i.e., CG and all-atom) by the AdResS method and the study of double-stranded DNA. Figure 1 shows the integration of different methodologies for the study of biomolecular complexes.

A first example of the power of CG descriptions is found in the work of Wettermann et al., where a bead-stick model is used to represent double strands of DNA under different ionic conditions and study their topological features. The analysis of the probability of knot formations for systems of hundreds of thousands of base pairs can be directly compared with data from nanopore experiments. Moreover, their analysis predicts a scenario where the knotting probability is extremely low and therefore, useful for setting up experiments where the knots are undesired.

Molecular modeling plays a crucial role in identifying binding motifs in large protein systems such as integrins, yet it is limited by system sizes. At cellular scale, significant conformational changes led to mechanotransduction, cell adhesion, differentiation, etc. In such context, the perspective article by Liu and Perez shows traditional routes for identifying collagen-like motifs that bind the I-domain of the *α*2*β*1 integrin, addressing their limitations and presenting alternative solutions by machine learning approaches (i.e., AlphaFold). Also, Oliva et al. propose another tool for protein structure modeling, Modelling eNvironment for Isoforms (MoNvIso), which aims to discover protein structures for genetic diseases. The method was tested on 70 proteins which correspond to 257 human isoforms. This procedure can handle large sets of proteins, but it can only model protein regions where structural templates are given. A comparison with AlphaFold supports validation of the MoNvIso approach for large search determination of protein-protein interactions.

Beyond the description and prediction of structures and interactions, the dynamic interplay between biological partners has a fundamental role in describing biomolecular complexes, especially when quantum and classical levels are required. The work by Jand et al. employed the multiscale approach, denoted as Adaptive Resolution Scheme (AdResS), to study the quantum delocalization in space of the water molecules during the aggregation process of two fullerene molecules. Using path-integral MD for the quantum part and all-atom/CG MD description for the classical region, they show the relevance of quantum effects in the free energy profiles with consequences in the formation of fullerene complexes.

Classical all-atom MD simulations are key for understanding biomolecular complexes, as they capture local conformations enabling the calculation of free energy profiles. Ramirez et al. elucidated the fingerprints of necroptotic pathways driven by the electrostatic interactions in protein-lipid complexes. Zargari et al. report on free energy calculation of protein-ligand by funnel metadynamics using all-atom MD. Their results successfully combine all-atom MD and enhanced sampling in docking studies. The work by Pham et al. combines statistical mechanics and MD simulation to obtain a better understanding of liquid-liquid phase transitions in cellular organelles. They propose several quantities to characterize the metastability regime, such as specific heat, surface tension, feature in molecular clusters, etc. They employed a Lennard-Jones model for the analysis of liquid-liquid transitions.

Similarly, Gomes et al. employed the Martini 3 force field combined with the GõMartini approach to capture the mechanical stability of bone sialoprotein binding protein in the early stages of Staphylococci infections, namely, the Bbp:Fgα complex. It required sampling significant conformational changes in protein complexes by means of steered CG and all-atoms MD simulations. The approach accurately described the stabilization mechanism of the Bbp:Fgα complex. The high force-loads present during the initial stages of bacterial infection stabilize β-sheet motifs in both proteins that, due to their position in the complex, cannot be peeled as in another bacterial system.

Multiscale modeling usually requires reintroducing all-atom details onto the CG trajectories to generate a complete atomistic picture. This task can be as challenging as the design of the CG model. Moreover, the simplification introduced by the CG simulation might generate conformations that have no correspondence in an all-atom representation. These important Research Topic are addressed by Hunkler et al., extending the Back-mapping Based Sampling (BMBS) to large systems, by applying it to the simulation of K48-linked tri-ubiquitin. The authors discuss how to correct the inaccuracies generated by the exploration in the CG level and distinguish relevant regions on a low-dimension projection of the conformational space. Their approach allows them to confidently access, with the all-atom resolution, parts of the conformation space that are very difficult or nearly impossible to explore by plain MD simulations.

Finally, a remarkable example of the capability of integrating coarse-grained representations, molecular modeling, and simulation techniques is provided by Stevens et al.. They combined a large volume of experimental data (i.e., cryoEM, cryoET, -omics data) to produce a model of a JCVI-syn3A cell. The integrative approach required the development of mesoscopic models integrated into the Martini 3 ecosystem by standard toolkits such as Polyply, Martinize2, and TS2CG. The length scale is nearly half a micrometer, with 561 million CG beads representing more than 6 billion atoms. The size and architecture of this cellular model represent a milestone in building a particle-based whole-cell model.

Certainly, subsequent multiscale simulations combining the techniques illustrated in this Research Topic will be instrumental in leading us to the next level of understanding and integration in cellular and structural biology.
